# Electroacupuncture ameliorates memory impairments by enhancing oligodendrocyte regeneration in a mouse model of prolonged cerebral hypoperfusion

**DOI:** 10.1038/srep28646

**Published:** 2016-06-28

**Authors:** Sung Min Ahn, Yu Ri Kim, Ha Neui Kim, Yong-Il Shin, Hwa Kyoung Shin, Byung Tae Choi

**Affiliations:** 1 Department of Korean Medical Science, School of Korean Medicine, Pusan National University, Yangsan 50612, Korea; 2 Korean Medical Science Research Center for Healthy-Aging, Pusan National University, Yangsan 50612, Korea; 3 Department of Rehabilitation Medicine, School of Medicine, Pusan National University, Yangsan 50612, Korea; 4 Division of Meridian and Structural Medicine, School of Korean Medicine, Pusan National University, Yangsan 50612, Korea

## Abstract

We modeled prolonged cerebral hypoperfusion in mice using bilateral common carotid artery stenosis (BCAS) and electroacupuncture (EA) stimulation was applied at two acupoints, Baihui (GV20) and Dazhui (GV14). In behavioral tests of memory, BCAS produced impairments in spatial and short-term memory in mice that were attenuated by therapeutic EA stimulation. Therapeutic use of EA in BCAS also enhanced oligodendrocyte (OL) differentiation from oligodendrocyte precursor cells (OPCs), in association with white matter improvements in the corpus callosum (CC). In PCR analyses of growth factor gene expression, significant positive changes in 3 genes were observed following EA stimulation in BCAS, and here we highlight alterations in neurotrophin-4/5 (NT4/5). We confirmed EA-mediated positive changes in the expression of NT4/5 and its receptor, tyrosine receptor kinase B (TrkB). Treatment of naïve and BCAS + EA animals with a selective TrkB antagonist, ANA-12, produced losses of myelin and cognitive function that were ameliorated by EA therapy. Moreover, following BCAS we observed an EA-dependent increase in phospho-activated CREB (a downstream mediator of NT4/5-TrkB signaling) in OPCs and OLs of the CC. Our results suggest that EA stimulation promotes the recovery of memory function following white matter injury via a mechanism that promotes oligodendrocyte regeneration and involves NT4/5-TrkB signaling.

Vascular dementia is the second most common form of dementia after Alzheimer’s disease^1^,^2^. Prolonged decreases in cerebral blood flow (hypoperfusion) produce white matter injury and are known to contribute to the pathophysiology of stroke and vascular dementia^3^. White matter damage attributed to small cerebral vessel disease is a critical component of vascular dementia^4^, in which cognitive impairment is referential to that seen in other cerebrovascular pathologies^2^,^3^. Cerebral white matter lesions are characterized by neuropathological changes such as myelin loss, axonal injury, and gliosis, and these pathological characteristics contribute to the deterioration of neurological function in animals and humans alike^5^,^6^,^7^,^8^.

In other states of brain injury such as stroke, endogenous neuroprotective responses including compensatory neurogenesis, angiogenesis, and remodeling mediate a balance between initial injury processes and endogenous repair processes^9^. Oligodendrocyte (OL) are the predominant cell type in white matter of the central nervous system (CNS). OLs mediate myelination as an essential process for the appropriate propagation of action potentials along axons^10^. Restoration of damaged white matter is therefore in part a function of oligodendrocyte precursor cell (OPC) differentiation into mature OLs in the adult brain^3^,^11^.

Although OPCs are capable of differentiation into mature, functional OLs in response to CNS injury, the capacity of these cells is not sufficient to prevent neurological and behavioral dysfunctions following injury^12^,^13^,^14^. Therefore, the development of therapies that enhance OL regeneration and myelination have profound utility for the treatment of CNS white matter injury. Electroacupuncture (EA) is a novel therapy that combines traditional acupuncture with modern electrotherapy. EA is currently in use as a complementary therapy for stroke and post-stroke rehabilitation in Korea^15^,^16^. In a model of ischemia, EA stimulation at appropriate acupoints with appropriate stimulation parameters was able to significantly alleviate neurological deficits^17^,^18^. EA enhances the proliferation and differentiation of neuronal progenitor cells under post-ischemic conditions and promotes neurological functional recovery from ischemia in rodents and humans^19^,^20^. Therefore, we hypothesized that EA may have similar benefits in a murine model of vascular dementia.

Cognitive impairments typical of vascular dementia and white matter injury are modeled in mice by prolonged cerebral hypoperfusion, which is achieved through the surgical induction of bilateral common carotid artery stenosis (BCAS)^4^,^21^,^22^,^23^,^24^. In the present work, we utilized the BCAS model to evaluate the ability of EA stimulation to attenuate memory impairments and white matter damage resultant from prolonged cerebral hypoperfusion. Specifically, we focused on the ability of EA to promote OL differentiation from OPCs, and investigated potential molecular mechanisms underlying this affect. A greater understanding of the molecular mechanisms and beneficial outcomes of EA in white matter dysfunction will aid the development of new therapeutic approaches for vascular dementia^22^.

## Results

### EA stimulation improves memory impairments associated with prolonged cerebral hypoperfusion

First, we investigated the specificity and relevance of the Baihui (GV20) and Dazhui (GV14) acupoints to BCAS-related memory impairment using the Morris water maze (MWM) test. On day 10 post-BCAS induction, BCAS + EA mice demonstrated a significantly shorter latency to locate platform compared to BCAS mice, whereas non-acupoint puncture or electrical stimulation had no effect (Fig. S1). Therefore, EA stimulation of the Baihui and Dazhui acupoints was utilized in subsequent experiments.

**Figure 1:**
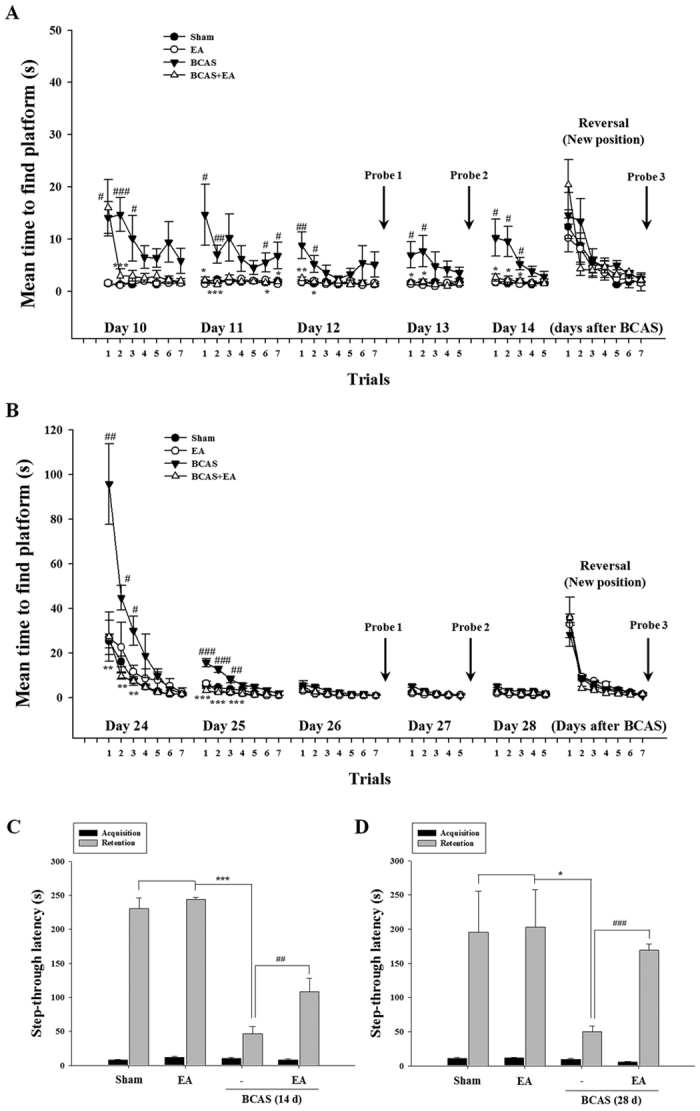
EA stimulation improves cognitive impairments following prolonged cerebral hypoperfusion. (**A,B**) Morris water maze test. Downward arrows indicate probe trials. ^#^*P* < 0.05, ^##^*P* < 0.01, and ^###^*P* < 0.001 vs. sham control; ^*^P < 0.05, ^**^P < 0.01, and ^***^P < 0.001 vs. BCAS group. (**C,D**) Passive avoidance test. ^*^P < 0.05 and ^***^P < 0.001 vs. sham control or EA group; ^##^*P* < 0.01 and ^###^*P* < 0.001 vs. BCAS group. Data expressed as the mean (±SEM).

In the MWM test for spatial learning and memory, BCAS mice demonstrated, on average, a longer latency to locate the platform than sham control mice, with an exception on days 26–28 post-BCAS induction. BCAS + EA mice demonstrated significantly lower latencies were observed on days 10–14 and 24–25 in BCAS + EA mice versus BCAS mice (Figs 1A,B and S2A,C). In probe testing conducted over 3 days, the percent time spent in proximity to the platform location was significantly increased on days 14 and 26 post-BCAS induction in groups receiving EA stimulation (Fig. S2B,D). In reversal trials conducted on days 14 and 28 post-BCAS induction, no clear differences were observed among groups (Fig. 1A,B).

In the passive avoidance test, no significant inter-group differences in the step-through latency were observed during acquisition trials, but a significant effect was observed in retention trials. On average, BCAS mice exhibited a significantly shorter step-through latency than sham control mice in retention trials conducted on days 14 and 28 post-BCAS induction; however, BCAS + EA mice demonstrated significantly longer latencies than BCAS mice in these same trials (Fig. 1C,D). Taken together, these behavioral results indicate that 7-day EA stimulation produces a beneficial effect on memory impairments resultant from prolonged cerebral hypoperfusion in mice.

### EA stimulation attenuates white matter injury following prolonged cerebral hypoperfusion

**Figure 2:**
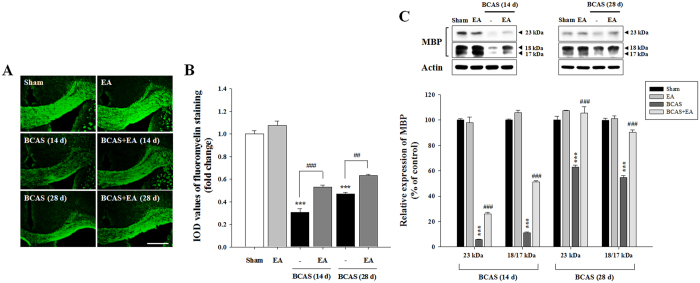
EA stimulation ameliorates white matter injury in the corpus callosum following prolonged cerebral hypoperfusion. (**A,B**) Representative fluoromyelin images at days 14 and 28 post-BCAS induction and histogram analyses. Scale bar = 400 μm. (**C**) Western blot images and histogram analyses of MBP expression at days 14 and 28 post-BCAS induction. The histogram indicates the mean (±SEM) for 3 independent experiments. ^***^P < 0.001 vs. sham control; ^##^*P* < 0.01 and ^###^*P* < 0.001 vs. BCAS group.

In order to visualize changes in white matter integrity following prolonged cerebral hypoperfusion, we evaluated fluoromyelin staining and myelin basic protein (MBP) expression in the corpus callosum (CC) following BCAS induction. In BCAS animals, a decrease in fluoromyelin staining (Fig. 2A,B) and a decreased MBP expression observed via Western blotting (Fig. 2C) was indicative of myelin loss due to white matter injury. Apparent myelin loss was most severe on day 14 post-BCAS induction. EA stimulation significantly enhanced fluoromyelin staining and MBP expression in the CC. These data suggest that EA stimulation produces significant attenuation of white matter injury following prolonged cerebral hypoperfusion.

### EA stimulation promotes the proliferation and differentiation of OLs following prolonged cerebral hypoperfusion

**Figure 3:**
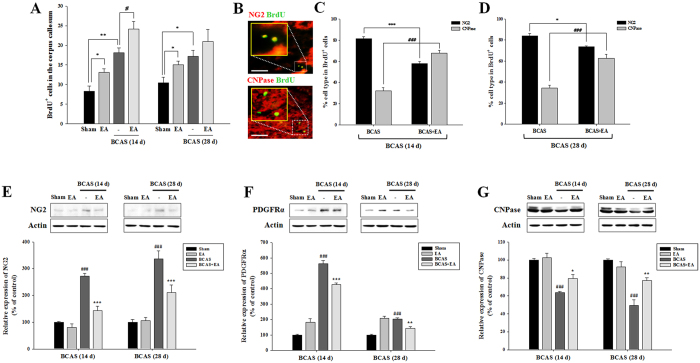
EA stimulation enhances OL differentiation from OPCs in association with recovered myelination in the corpus callosum. (**A**) Numbers of BrdU-positive (BrdU^+^) cells at days 14 and 28 post-BCAS induction. Data expressed as the mean (±SEM). ^*^P < 0.05 and ^**^P < 0.01 vs. sham control; ^#^*P* < 0.05 vs. BCAS group. (**B**) Double immunofluorescent staining for BrdU and NG2 (a marker for OPCs) or CNPase (a marker for OLs) in the sham control condition. Scale bar = 50 μm. (**C,D**) Ratio of BrdU/NG2 or BrdU/CNPase double-labeled cells in total BrdU^+^ cells at days 14 (**C**) and 28 (**D**) post-BCAS induction. Data expressed as the mean (±SEM). ^*^P < 0.05, ^***^P < 0.001, or ^###^*P* < 0.001 vs. BCAS group. (**E–G**) Western blotting analysis of OPCs protein NG2 (**E**) or PDGFRα (**F**), and OLs protein CNPase (**G**) from corpus callosum lysates. Data expressed as the mean (±SEM). ^###^*P* < 0.001 vs. sham control; ^*^P < 0.05, ^**^P < 0.01, and ^***^P < 0.001 vs. BCAS group.

5-bromo-2′-deoxyuridine (BrdU; 50 mg/kg/day, 7 days, i.p.) during EA stimulation sessions and the number of BrdU-positive cells in the CC was measured post-mortem. BrdU incorporation was increased in the CC of BCAS + EA mice, and this effect was pronounced on day 14 post-BCAS induction (Fig. 3A). A fewer number of double-labeled BrdU^+^/NG2^+^ cells (indicative of proliferating OPCs) were observed in BCAS + EA mice versus untreated BCAS mice. Conversely, a larger number of double-labeled BrdU^+^/CNPase^+^ cells (indicative of newly differentiated OLs) was observed in the CC of BCAS + EA mice versus untreated BCAS mice (Fig. 3B–D). In corroboration of our immunohistochemical observations, Western blotting of OPC protein markers neural/glial antigen 2 (NG2) and platelet-derived growth factor receptor-α (PDGFRα) were significantly decreased and the OL marker 2,3-cyclic nucleotide-3-phosphodiesterase (CNPase) was significantly increased in the CC of BCAS + EA mice versus BCAS mice (Fig. 3E–G).

The subventricular zone (SVZ) is a critical site for endogenous preservation of OPCs. In our studies, we observed no significant changes in expression of the proliferative marker Ki67 at days 14 and 28 post-BCAS induction between BCAS and sham control groups; however, Ki67-positive cells were co-localized with PDGFRα between the SVZ and CC, suggesting that white matter repair is also mediated by OPCs migrating from the SVZ to the CC (Fig. S3A,B). These results suggest that EA stimulation may enhance development of OL-lineage cells by promoting the differentiation of OPCs into mature OLs.

### EA stimulation upregulates NT4/5 gene after prolonged cerebral hypoperfusion

**Figure 4:**
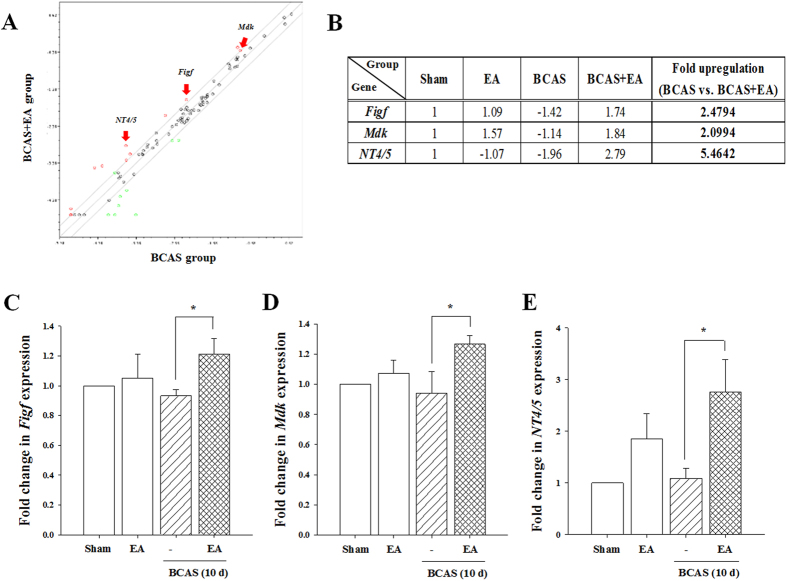
Gene expression profiles in corpus callosum extracts from BCAS and BCAS + EA groups. Gene expression profiles were evaluated using the Mouse Growth Factors RT^2^ profiler PCR array. (**A**) Scatter plot summarizing gene expression analyses. The middle diagonal gray line indicates 1-fold change (no change of gene expression between both groups). Circles above this gray line indicate an increase gene expression, and circles below indicate a decrease in expression, BCAS + EA versus BCAS group. The two outer gray lines indicate a 2-fold change. The 11 red circles represent genes that showed a >2-fold incrsease in expression in the BCAS + EA versus BCAS group comparison. 3 genes (*Figf*, *Mdk*, and *NT4/5*) are indicated by arrows. (**B**) The table shows select genes (*Figf*, *Mdk*, and *NT4/5*), that demonstrated differential expression between the BCAS and BCAS + EA groups. Numbers in bold indicate upregulated genes. (**C–E**) Fold change in expression of the 3 selected genes as detected by quantitative real-time PCR analysis. *Figf*, c-fos induced growth factor; *Mdk*, midkine; *NT4/5*, neurotrophin-4/5. Data expressed as the mean (±SEM). ^*^P < 0.05 vs. BCAS group.

Growth factor gene expression in the CC was evaluated immediately following the final EA stimulation session. Using the arbitrary cutoff of >2-fold change, we observed a significant positive effect of EA stimulation on 11 genes (Fig. 4A), and results were confirmed using real-time polymerase chain reaction (PCR). We noted a significant increase in the expression of 3 genes (*Figf* (c-fos induced growth factor), *Mdk* (midkine), and *NT4/5* (neurotrophin-4/5) in BCAS + EA versus BCAS mice (Fig. 4C–E). In particular, *NT4/5* gene was prominently upregulated (5-fold in PCR array) in the CC of BCAS + EA versus BCAS mice (Fig. 4B,E). These data suggest that EA stimulation enhances the expression of specific growth factors-in particular, NT4/5-following prolonged cerebral hypoperfusion.

### EA stimulation promotes NT4/5 and TrkB expression following prolonged cerebral hypoperfusion

**Figure 5:**
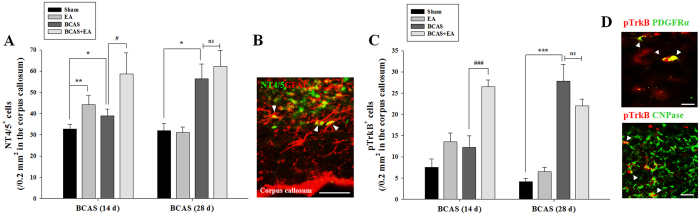
EA stimulation promotes NT4/5-TrkB expression in the corpus callosum following prolonged cerebral hypoperfusion. (**A**) Numbers of NT4/5-positive (NT4/5^+^) cells at days 14 and 28 post-BCAS induction. Data expressed as the mean (±SEM). ^*^P < 0.05, and ^**^P < 0.01 vs. sham control; ^#^*P* < 0.05 vs. BCAS group. (**B**) NT4/5/GFAP double-labeled cells (arrowheads) in the BCAS + EA group at day 14. Scale bar = 50 μm. (**C**) Numbers of pTrkB-positive (pTrkB^+^) cells at days 14 and 28 post-BCAS induction. Data expressed as the mean (±SEM). ^***^P < 0.001 vs. sham control; ^###^*P*<0.001 vs. BCAS group. (D) pTrkB-positive cells with PDGFRα (a marker for OPCs) or CNPase (a marker for OLs) (arrowheads) in the BCAS + EA group at day 14 post-BCAS induction. Scale bar = 25 μm.

The expression of NT4/5 and its receptor, tyrosine receptor kinase B (TrkB), were evaluated in the CC. The number of NT4/5-positive cells was increased in BCAS + EA versus BCAS mice at 14 days post-BCAS induction, and this immunoreactivity was primarily observed in GFAP-positive cells (i.e., astrocytes) (Fig. 5A,B). The number of pTrkB-positive cells showed a pattern of distribution similar to that of NT4/5. Moreover, the expression of pTrkB was largely co-localized with PDGFRα- or CNPase-positive cells in the CC (Fig. 5C,D). Conversely, the number of NT4/5-positive cells in the SVZ was increased in both BCAS + EA and BCAS groups versus the sham control group, but no significance was detected between the BCAS + EA and BCAS groups. In the SVZ, NT4/5 expression was primarily co-localized with neuronal nuclei (NeuN)-positive (i.e., neurons) (Fig. S4A–C). The number of pTrkB-positive cells was slightly increased in BCAS + EA versus BCAS mice, but the effect was non-significant (Fig. S4D–F). These data indicate that EA stimulation in BCAS mice promotes the development of OL-lineage cells in the CC through a mechanism that involves NT4/5-TrkB expression.

### TrkB antagonist ANA-12 inhibits beneficial effects of EA

**Figure 6:**
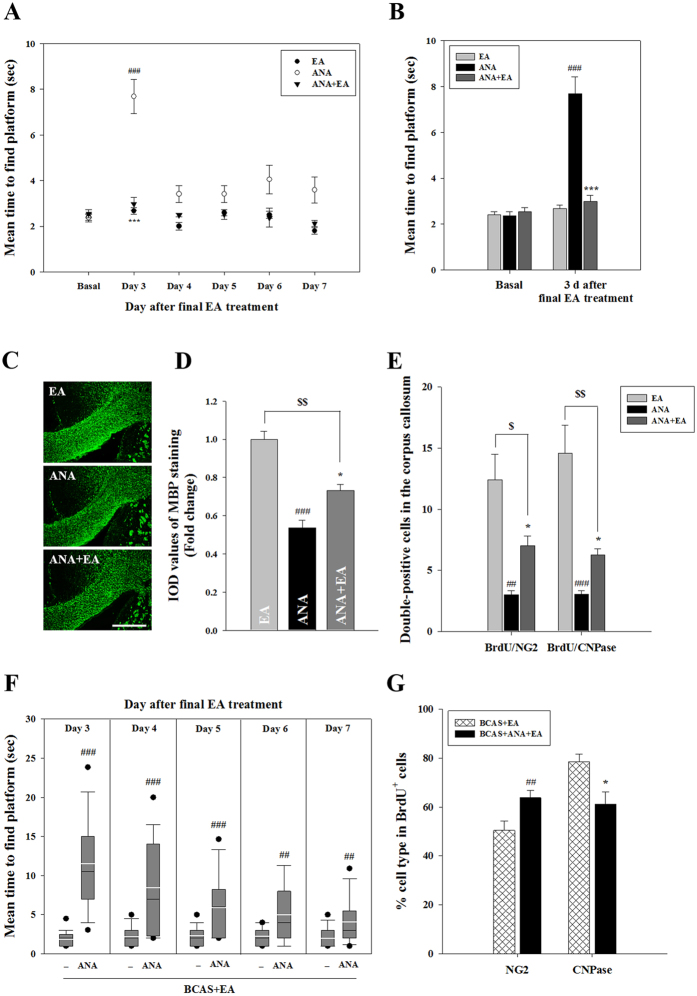
The effect of TrkB antagonist ANA-12 on the beneficial effects of EA. (A-E) The effect of TrkB antagonist ANA-12 in naïve animals. (**A,B**) Morris water maze test. Mean time to locate the platform in the ANA-12 group was significantly reduced by EA treatment 3 days following the cessation of therapy. (**C,D**) Representative MBP-stained images of the corpus callosum and MBP histogram analysis. Data expressed as the mean (±SEM). ^###^*P* < 0.001, or ^$$^*P* < 0.01 vs. EA group; ^*^P < 0.05 vs. ANA-12 group. Scale bar = 400 μm. (**E**) Double immunofluorescent staining of BrdU and NG2 or CNPase in the corpus callosum. Data expressed as the mean (±SEM). ^##^*P* < 0.01, ^###^*P* < 0.001, ^$^*P* < 0.05, and ^$$^*P* < 0.01 vs. EA group; ^*^P < 0.05 vs. ANA-12 group. (**F,G**) The effect of TrkB antagonist ANA-12 in BCAS + EA group. (**F**) Morris water maze test. ANA-12-treated BCAS + EA mice showed a significant increase in mean time to locate the platform compared to BCAS + EA mice. The box plot indicates the medians (black lines), the mean (white lines), 25th–75th percentiles (box), and 5th–95th percentiles (whiskers). (**G**) Double immunofluorescent staining of BrdU and NG2 or CNPase in the corpus callosum. Data expressed as the mean (±SEM). ^##^*P* < 0.01, ^###^*P* < 0.001 and ^*^P < 0.05 vs. BCAS + EA group.

We employed a potent, specific TrkB antagonist, ANA-12 (0.5 mg/kg/day, 7 days, i.p.), in order to evaluate the functional significance of changes in NT4/5 and TrkB expression following EA stimulation. In the MWM, ANA-12 treatment significantly prolonged the average latency to locate the platform 3 days after the cessation of treatment. Notably, EA stimulation attenuated ANA-12-induced cognitive deficits in the MWM test (Fig. 6A,B). Consistent with the observed impairment of spatial memory, ANA-12 treatment produced a reduction in fluoromyelin staining in the CC, and this effect was in part recovered in the ANA-12+EA treatment group. Moreover, ANA-12 treatment reduced the number of double-labeled BrdU^+^/NG2^+^ OPCs or BrdU^+^/CNPase^+^ OLs, and this reduction was also attenuated in the ANA-12+EA group (Fig. 6C–E). To confirm that EA mediated the regeneration of OLs through the NT4/5-TrkB signaling pathway in BCAS mice, we performed additional experiments investigating the effect of ANA-12 on BCAS + EA mice. In the MWM test, ANA-12 pretreatment in BCAS + EA mice increased the average latency to locate the platform compared to control BCAS + EA mice (Fig. 6F). Consistent with these behavioral observations, ANA-12 pretreatment in the BCAS + EA group also reduced the number of double-labeled BrdU^+^/CNPase^+^ OLs compared to the control BCAS + EA group, whereas the number of double-labeled BrdU^+^/NG2^+^ OPCs increased (Fig. 6G). These data suggest that NT4/5-TrkB signaling may be involved in the mechanism of EA-mediated improvement of white matter integrity.

### EA stimulation promotes CREB phosphorylation following prolonged cerebral hypoperfusion

**Figure 7:**
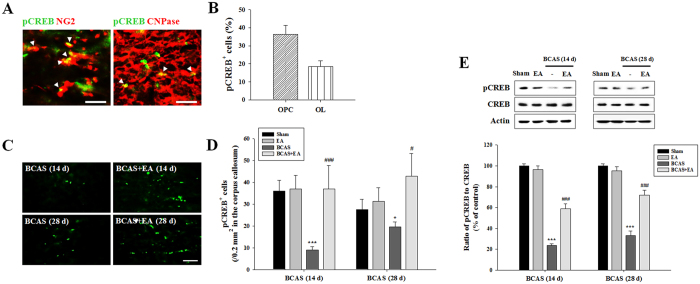
EA stimulation promotes CREB activation in the corpus callosum following prolonged cerebral hypoperfusion. (**A,B**) Double immunofluorescent staining of pCREB and NG2 or CNPase in the sham control condition and histogram analysis of pCREB-positive cells also identified as OPCs and OLs. Arrowheads indicate double-labeled cells. Scale bar = 25 μm. (**C,D**) Numbers of pCREB-positive (pCREB^+^) cells at days 14 and 28 post-BCAS induction and pCREB histogram analysis. Scale bar = 100 μm. (**E**) pCREB Western blot images. The histogram indicates the mean (±SEM) of 3 independent experiments. Data expressed as the mean (±SEM). ^*^P < 0.05, and ^***^P < 0.001 vs. sham control; ^#^*P* < 0.05, or ^###^*P* < 0.001 vs. BCAS group.

Cyclic AMP response element binding protein (CREB) phosphorylation occurs downstream of neurotrophic signaling. In sham animals, pCREB was co-localized with NG2 and CNPase in 36.4% and 18.4% of total pCREB-positive cells in the CC, respectively (Fig. 7A,B). At day 14 or 28 post-BCAS induction, the total number of pCREB-positive cells was significantly decreased in both BCAS-operated groups versus the sham control group. Importantly, pCREB-positive cells were significantly increased in the BCAS + EA versus BCAS groups (Fig. 7C,D). Western blot analysis confirmed a decrease in the expression of pCREB in the BCAS group, and a recovery of pCREB expression the BCAS + EA group, where pCREB expression was significantly increased in BCAS + EA versus BCAS groups (Fig. 7E). These data suggest that EA stimulation may enhance the phosphorylative activation of CREB in order to produce the observed enhancement of OPC differentiation into mature OLs following prolonged cerebral hypoperfusion.

## Discussion

Herein we provide the first evidence that, following prolonged cerebral hypoperfusion, EA therapy rescues cognitive impairment and ameliorates white matter damage in the CC by enhancing OL differentiation. Furthermore, we provide data to support the hypothesis that EA promotes the OL cell lineage through a mechanism that involves neurotrophic growth factor signaling, and specifically, NT4/5. Our findings indicate that targeting OL differentiation with therapeutic EA stimulation may represent a useful complementary approach to the treatment of white matter injuries such as vascular dementia.

White matter injury caused by a prolonged decrease in cerebral blood flow is a prominent characteristic of vascular dementia^1^,^4^,^18^. An insufficient blood supply to the brain produces a loss of myelin and subcortical vascular dementia, which is a major subtype of vascular dementia^4^,^6^,^7^,^25^. Surgical narrowing of the bilateral common carotid arteries with microcoils produces white matter pathology in mice that is both highly reproducible and relevant to the clinical manifestation of vascular dementia^22^,^26^.

Accordingly, we employed BCAS as a murine model of prolonged cerebral hypoperfusion in order to investigate the relationship between behavioral cognitive deficits and white matter pathology (i.e., myelin integrity) in the CC. A previous study by our group showed that EA stimulation promotes post-ischemic stroke functional recovery by enhancing the proliferation and differentiation of neuronal stem cells in mice^19^. Based on this findings, we employed EA stimulation at a low frequency of 2 Hz (output voltage: 2 V) in a therapeutic modality and predicted the observation of similar beneficial effects in the BCAS model. On day 4 post-BCAS induction, we initiated 7 consecutive days of EA stimulation and found that EA promoted the functional recovery of spatial and short-term memory deficits. We did observe compensatory mechanisms of recovery on days 26–28 post-BCAS induction, and this effect has been characterized in a previous study^26^. Consistent with behavioral data, EA stimulation also significantly improved fluoromyelin staining and MBP expression in the CC, and these data were interpreted to be enhancements in white matter integrity. Improvements in white matter pathology are strongly correlated with the recovery of memory deficits in most disorders of the brain^9^. Therefore, the ability of EA therapy to target white matter may have particular implications for the treatment of white matter injury-associated memory impairments.

Myelin-forming OLs are the predominant cell type in white matter, and these cells essentially maintain the myelin sheath and axonal integrity in the CNS^10^. OL loss and demyelination are cellular hallmarks of white matter injury in human diseases^27^. White matter injury induces residual OPC proliferation, migration to the site of injury, and differentiation into mature OLs in order to restore myelin losses^13^,^14^. Therefore, neurological disorders produce a balance of initial injury characteristics and endogenous repair engagement^3^,^5^.

In our study, an increase in BrdU incorporation in the CC was indicative of an increase in the proliferation of neuronal progenitor cells. However, this increase in BrdU-positive cells in the CC was not directly indicative of the proliferation and differentiation of specific cells such as OPCs or OLs. BrdU co-localization with cellular markers indicated that EA stimulation reduced the number of new OPCs and increased the number of newly differentiated OLs in the CC following BCAS, suggesting that EA stimulation promotes the differentiation of OPCs into OLs in BCAS mice. That is, this effect of EA can be explained by enhancement of OPC-to-OL differentiation in the BCAS + EA group, while EA stimulation of sham group only enhanced the proliferation of residual OPCs. Therefore, our findings may be extended to suggest that EA stimulation is likely to promote OL differentiation after prolonged cerebral hypoperfusion in patients.

Neural progenitors in the SVZ represent an important endogenous source of OPCs for the generation of new myelinating OLs for neighboring white matter tracts including the CC^28^,^29^,^30^. However, we did not observe significant changes in Ki67, a marker of cellular proliferation, in the SVZ between BCAS + EA and BCAS groups. However, migrating progenitor cells co-labeled with PDGFRα and Ki67 double-positive were observed between the SVZ and CC. These data indicate that migrating OPCs may be relevant to white matter injury and recovery.

Mature myelinating OLs are continuously differentiated from local OPCs residing in the brain parenchyma in response to demyelinating lesions. Therefore, parenchymal OPCs do not need to migrate extensively following white matter injury in order restore myelin sheaths in the neighboring area^28^. Given that the differentiation of OPCs into mature myelinating OLs is a critical step for myelination, it may be inferred that, rather than promoting OPC proliferation distant from myelin injury in the SVZ. EA stimulation can be explained by, EA enhances the differentiation of OPCs local to injury (e.g., in the parenchyma of the CC).

Endogenous repair mechanisms in the CNS including oligodendrocyte regeneration are associated with growth factor signaling^31^. An increase in the gene expression of growth factors are observed in response to EA, including brain-derived neurotrophic factor (BDNF), nerve growth factor (NGF), NT3, NT4, NT5, and associated receptors^32^,^33^. Notably, EA treatment has been previously reported to increase the expression of NT3 and promote OL-like cell differentiation in association with functional improvements^34^. In our work, we demonstrate EA-associated alterations in several genes including *Figf* (vascular endothelial growth factor), *Mdk* (neurite growth-promoting factor 2), and most significantly the neurotrophic factor family *NT4/5*. Whereas NT3 has a well-established role in axonal regeneration, OPCs differentiation, and central remyelination following white matter injury^35^,^36^, little is known about the role of NT4/5 in the development of OL lineage cells in response to white matter injury. In the present study, we identify elevations in both NT4/5 and its receptor TrkB in the CC but not the SVZ following EA stimulation in BCAS mice. NT4/5 was detected in astrocytes, and TrkB was identified in OPCs and mature OLs. These cumulative findings corroborate the hypothesis that EA selectively promotes OL regeneration at sites local to white matter injury (i.e., the CC) rather than at distant sites (e.g., the SVZ).

The functional relevance of NT4/5-TrkB signaling to the beneficial effect of EA in BCAS was evaluated by the administration of a specific TrkB antagonist, ANA-12, both alone and in combination with EA stimulation in sham and BCAS mice. ANA-12 alone produced a temporary decline in spatial memory performance that was associated with myelin loss and repressed OPC proliferation/differentiation. Importantly, these effects were rescued by EA stimulation in the ANA-12 + EA group. Additionally, ANA-12 suppressed the enhancement of OL regeneration promoted by EA stimulation after BCAS, indicating that NT4/5-TrkB signaling is relevant to EA-mediated enhancements of OL differentiation in BCAS. Further studies are required to evaluate whether activation of NT4/5-TrkB signaling is critical to the beneficial actions of EA therapy in white matter injury following prolonged cerebral hypoperfusion.

Diverse extracellular stimuli elicit neurotrophin signaling and NPC proliferation, survival, and differentiation through phosphorylative activation of the transcription factor CREB^37^,^38^. BDNF and NT4/5 activation of TrkB lead to the activation of CREB in pathological conditions^37^. In our study, the expression of active phosphorylated CREB was observed in both OPCs and mature OLs under normal conditions, and EA increased this pattern of pCREB expression in BCAS mice. These data indicate that the induction of CREB signaling, possibly via NT4/5-TrkB signaling, underlies OL differentiation and white matter injury repair following prolonged cerebral hypoperfusion.

Our current body of work demonstrates that therapeutic EA stimulation improves functional recovery prolonged cerebral hypoperfusion-induced cognitive impairment by promoting OL differentiation. We also demonstrate for the first time that NT4/5-TrkB signaling plays a role in OL response to white matter injury. However, we note two main limitations of our study: first, it is important to note that other growth factors may play a role in the beneficial actions of EA stimulation, as several growth factors were positively regulated by EA therapy. We selected NT4/5 for further investigation based the observed effect size (5-fold change between BCAS + EA and BCAS groups). However, BDNF also activates TrkB and plays a crucial role in OL proliferation, differentiation, and myelination^39^,^40^. Therefore, we cannot exclude the possibility that the effects of EA are in part mediated by BDNF-TrkB signaling or other neurotrophic factors.

Secondly, it is vital to note that mature OLs are vulnerable to modest blood flow reductions^41^, and therefore NT4/5-TrkB signaling may promote parenchymal OPC and OL survival instead of or in addition to the promotion of OL myelin regeneration. Parenchymal OPCs and OLs are damaged by oxidative stress or inflammation under conditions of cerebral hypoperfusion^42^. Our results indicate the activation CREB signaling in OPCs and OLs, but it is important to note that this activation may also serve to promote the gene expression of neuroprotective molecules and anti-apoptotic pathways^14^,^38^ as a component of the beneficial effect mediated by EA.

While the neurological repair mechanisms initiated by EA therapy require additional investigation, our study provides critical *in vivo* evidence that EA improves white matter pathology and associated cognitive impairments following prolonged cerebral hypoperfusion in mice, and for the first time implicates the NT4/5-TrkB pathway in OL-mediated regeneration. Accordingly, EA represents a novel and therapeutically relevant option for assisting functional recovery in vascular dementia and other forms of white matter injury.

## Methods

### Animals

Male C57BL/6 mice aged 11 weeks were obtained from Dooyeol Biotech (Seoul, Korea). Mice were housed at 22 °C on a 12-h light/dark cycle and were fed a commercial diet and allowed tap water *ad libitum* for the duration of the study. All experiments were approved by the Pusan National University Animal Care and Use Committee in accordance with the National Institutes of Health Guidelines (approval number PNU-2015-0781).

### Prolonged cerebral hypoperfusion model

For the induction of chronic cerebral hypoperfusion, 2 microcoils with a 0.18 mm internal diameter (Sawane Spring Co, Hamamatsu, Japan) were surgically applied to produce bilateral stenosis of the common carotid artery (BCAS). Briefly, mice were anesthetized with isoflurane (2% induction and maintenance; Choongwae, Seoul, Korea) in 80% N_2_O and 20% O_2_, and a rectal temperature of 36.5–37.5 °C was maintained using a Panlab thermostatically controlled heating mat (Harvard Apparatus, Holliston, MA, USA). The operation time was approximately 20 minutes per mouse, and the interval between the 2 microcoils was 5 min. Sham control mice underwent an identical procedure without microcoil application. All experimental procedures were performed in a blinded and randomized fashion.

### EA Stimulation

Animal were anesthetized with isoflurane to minimize stress during EA stimulation sessions. Two bilateral stainless steel needles (0.18 mm diameter) were inserted to a depth of approximately 2 mm at the murine acupoints corresponding to Baihui (GV20, the midpoint of the line connecting the apexes of both ears on the parietal bone) and Dazhui (GV14, the posterior midline in the depression below the spinous process of the seventh cervical vertebra) and were connected to a Grass S88 electrostimulator (Grass Instrument Co., West Warwick, RI, USA). EA stimulation lasted for 20 min at a frequency of 2 Hz and output voltage was set at 2 V in accordance with previous studies^16^,^17^,^19^. EA treatment was initiated 4 days post-BCAS induction, and was administered once daily for 7 consecutive days. Sham subjects in the non-EA groups received isoflurane anesthesia for 20 min once daily for 7 consecutive days. As a control for the Baihui and Dazhui acupoints, needles were inserted into non-acupoints 1 mm lateral point to specific acupoints with or without electric stimulation^17^.

### BrdU Labeling

BrdU (AbD Serotec, Oxford, UK) is a synthetic thymidine analog that chelates DNA during the S-phase of cellular division. For cell proliferation and differentiation analyses, mice received intraperitoneal (i.p.) injections of BrdU (50 mg/kg) once daily for 7 consecutive days during EA stimulation.

### Behavioral assessments

In experiment 1, the MWM test was performed in order to evaluate spatial learning and memory. Acquisition training for the MWM test was performed for 5 consecutive days (7 trials per day) before the induction of BCAS. Baseline measurements were observed prior to training. A tank with a diameter of 100 cm and a height of 50 cm was used for MWM testing. The tank was filled with water and a platform was placed 0.5 cm beneath the surface of the water. Trials lasted for 90 s or until the mouse located the platform. Experimental results were recorded using SMART, version 2.5.18 (Panlab, S.L.U., Barcelona, Spain). Probe trial sessions (probe trials 1 and 2) took place on days 12–13 and 26–27 post-BCAS induction. In each probe trial, the platform was removed and memory performance was considered proportional to the percentage of spent time within a 20 cm diameter annulus surrounding the original platform location. Probe trials were followed by 5 additional trials in which the platform was replaced in its original location. Reversal trials were conducted in the afternoon on days 14 and 28 post-BCAS induction. In reversal trials, the platform was moved to a new location and the ability of mice to locate and remember the new platform position was measured in 7 subsequent trials. A probe trial (probe 3, days 14 and 28 post-BCAS induction) began 10 min after the reversal trials.

In experiment 2, a passive avoidance test was used to assess short-term memory function. The chamber consisted of one illuminated compartment and one dark compartment separated by an automatic guillotine door (Med-Associates, Inc., St. Albans, VT, USA). All animals received daily training in passive avoidance chambers for 3 consecutive days prior to acquisition and retention trials. During acquisition trials, mice who crossed into the dark compartment received a 0.5 mA electric foot shock that lasted 3 s. 24 h later, a retention trial was administered by placing the mice in the light compartment and recording the step-through latency (i.e., the latency to crossing into the dark compartment), during which time no shocks were administered. Maximal testing time was 600 s. Animals who failed to enter the dark compartment within 600 s were assigned a maximum test latency score of 600 s. Results of the experiment were recorded using MED-PC software interfaced with the test apparatus.

### Fluoromyelin stain

Mouse brain was removed and post-fixed in 4% paraformaldehyde in PBS for 24 h at 4 °C before cryoprotection in 30% sucrose. Frozen 20-μm sections were rinsed in PBS for at least 20 min. Sections were then incubated with FluoroMyelin^TM^ Green fluorescent myelin stain (1:300, Molecular probes, Eugene, OR, USA) for 20 min at room temperature, and then slides were mounted using mounting medium (Vector Laboratories, Inc., Burlingame, CA, USA). Semi-quantification of the intensity of fluoromyelin staining was conducted using 10× magnification images that were captured using a fluorescence microscope (Carl Zeiss Imager M1, Carl Zeiss, Inc., Gottingen, Germany).

### Western blot analysis

Tissue samples of corpus callosum were homogenized with lysis buffer containing 200 mM Tris (pH 8.0), 150 mM NaCl, 2 mM EDTA, 1 mM NaF, 1% NP40, 1 mM PMSF, 1 mM Na_3_VO_4_, and a protease inhibitor cocktail. Equal amounts of proteins were separated on a 7.5–12% sodium dodecyl sulfate-polyacrylamide gel using electrophoresis (SDS-PAGE) and then transferred to a nitrocellulose membrane (Whatman GmbH, Dassel, Germany). Membranes were blocked with 5% non-fat dry milk dissolved in PBST for 1 h. After washing, the membrane was probed with primary antibodies against MBP (Abcam, Cambridge, UK), NG2 (Abcam), PDGFRα (BD Biosciences, San Diego, CA, USA), CNPase (Abcam), CREB (Cell Signaling Technology, Danvers, MA, USA), phospho-CREB (pCREB, Ser133; Cell Signaling), or actin (Sigma-Aldrich, St. Louis, MO, USA) overnight at 4 °C on a shaker. Membranes were then incubated with the appropriate horseradish peroxidase-conjugated secondary antibodies for 1 h. All specific bands were visualized using an enhanced chemiluminescence system (Pierce Biotech, Rockford, IL, USA) and imaged using an Image Quant LAS-4000 imaging system (GE Healthcare Life Science, Uppsala, Sweden).

### Immunohistochemistry

Frozen 20-μm thick sections were incubated in a blocking buffer (PBS containing 5% normal serum and 0.3% Triton X-100) for 1 h at room temperature. Sections were then incubated overnight in PBS at 4 °C with one the following primary antibodies: BrdU (AbD Serotec, Oxford, UK), NG2 (Abcam), CNPase (Abcam), pCREB (Santa Cruz Biotechnology, Santa Cruz, CA, USA), MBP (Abcam), PDGFRα (BD Biosciences), NT4/5 (Santa Cruz Biotechnology), NeuN (Millipore Corporation, Billerica, MA, USA), or phospho-TrkB (Tyr515; Abcam). After washing with PBS, sections were incubated with an appropriate fluorescent secondary antibody (Vector Laboratories, Inc.) for 2 h at room temperature in the dark and then washed thrice with PBS. Subsequently, slides were mounted using mounting medium (Vector Laboratories, Inc.) and images were captured using a fluorescence microscope (Carl Zeiss, Inc.).

### Gene expression profiling

After the final EA stimulation session, animals were sacrificed and total RNA was isolated from the CC of brains using the RNeasy Mini Kit (Qiagen, Hilden, Germany) and transcribed to complementary DNA (cDNA) using the RT^2^ First Strand Kit (Qiagen). cDNA was pooled from all experimental conditions, and PCR array analyses were performed according to manufacturer specifications. A diluted first-strand cDNA synthesis reaction mixture was used for real-time PCR using RT^2^ SYBR Green ROX FAST Mastermix (Qiagen) and the Rotor-Gene Q Real-Time PCR detection system (Qiagen). Eighty-four target genes were analyzed using the Mouse Growth Factors RT^2^ Profiler PCR array in Rotor-Disc 100 format (PAMM-041Z, Qiagen). Differences of gene expression were analyzed using Qiagen’s web-based RT^2^ Profiler PCR Array Data Analysis software, version 3.5 (http://pcrdataanalysis.sabiosciences.com/pcr/arrayanalysis.php). Data were normalized using multiple housekeeping genes and analyzed by comparing the 2^−ΔCT^ of normalized data.

### Real time polymerase chain reaction

Real-time PCR (qPCR) was performed using first strand cDNA and specific primers. Total RNA was isolated from brain tissue using the RNeasy Mini Kit (Qiagen) according to the manufacturer’s instructions. Following isolation, 3 μg of total RNA was reverse transcribed to cDNA using the RT^2^ First Strand Kit (Qiagen). The amplification step was performed using the Rotor-Gene Q Real-Time PCR detection system (Qiagen) in 10 μl reaction mixtures containing 2 μl diluted DNA template, 5 pmol of each primer, and 5 μl 2X SYBR^®^ Premix Ex Taq^TM^ (Takara BIO, Otsu, Shiga, Japan). Glyceraldehyde-3-phosphate dehydrogenase (GAPDH) was used as a housekeeping control gene and all experiments were performed in triplicate for each sample. Gene expression was quantified by comparing the 2^−ΔCT^ of normalized data. All primers for SYBR green reactions are listed below.

*Figf* forward: 5′-CGAAGAGGGTGTGATGTGTATG-3′

*Figf* reverse: 5′-CTGGAGGTAAGAGTGGTCTTCT-3′

*Mdk* forward: 5′-TGGAGCCGACTGCAAATAC-3′

*Mdk* reverse: 5′-CTCTCTGGCCTCCTGACTTA-3′

*NT4/5* forward: 5′-CAAGGCTAAGCAGTCCTATGT-3′

*NT4/5* reverse: 5′-CAGTCATAAGGCACGGTAGAG-3′

*GAPDH* forward: 5′-CACCATCTTCCAGGAGCGAG-3′

*GAPDH* reverse, 5′-CCTTCTCCATGGTGGTGAAGAC-3′

### TrkB antagonist studies

Mice were randomly distributed into EA, ANA-12, and ANA-12+EA treatment groups (n = 7 per group). For blockade of TrkB signaling, the specific TrkB antagonist ANA-12 (0.5 mg/kg/day, 7 days, i.p.; Tocris Bioscience, Bristol, UK)^43^ was administered 1 h prior to each EA session. EA was applied once daily for 7 consecutive days and MWM tests were performed 5 days after treatment initiation and 3 days after treatment completion. Experimental results were recorded using SMART, version 2.5.18 (Panlab S.L.U.). Mice were sacrificed and tissues were analyzed for MBP expression and BrdU incorporation after the final MWM test.

### Statistical Analyses

All data were expressed as the mean (±SEM) and analyzed using SigmaStat statistical software version 11.2 (Systat Software, San Jose, CA, USA). Statistical comparisons were performed using a one-way analysis of variance (ANOVA) with repeated measures and Tukey’s post hoc test of least significant difference. A *P*-value < 0.05 was interpreted as statistically significant.

## Additional Information

**How to cite this article**: Ahn, S. M. *et al*. Electroacupuncture ameliorates memory impairments by enhancing oligodendrocyte regeneration in a mouse model of prolonged cerebral hypoperfusion. *Sci. Rep*. **6**, 28646; doi: 10.1038/srep28646 (2016).

## Supplementary Material

Supplementary Information
