# Supplementary material for: Electroacupuncture ameliorates memory impairments by enhancing oligodendrocyte regeneration in a mouse model of prolonged cerebral hypoperfusion
Source: Sci Rep. 2016 Jun 28;6:28646. doi: 10.1038/srep28646 (PMC4923909; doi:10.1038/srep28646)

Supplementary Information

Electroacupuncture ameliorates memory impairments by enhancing oligodendrocyte regeneration in a mouse model of prolonged cerebral hypoperfusion

Sung Min Ahn[1,2], Yu Ri Kim[1], Ha Neui Kim[1,2], Yong-Il Shin[3], Hwa Kyoung Shin[1,2,4] & Byung Tae Choi[1,2,4,*]

[1]Department of Korean Medical Science, School of Korean Medicine, Pusan National University, Yangsan 50612, Korea

[2]Korean Medical Science Research Center for Healthy-Aging, Pusan National University, Yangsan 50612, Korea

[3]Department of Rehabilitation Medicine, School of Medicine, Pusan National University, Yangsan 50612, Korea

[4]Division of Meridian and Structural Medicine, School of Korean Medicine, Pusan National University, Yangsan 50612, Korea

*Corresponding author: Dr. Byung Tae Choi, Department of Korean Medical Science, School of Korean Medicine, Pusan National University, Yangsan 50612, Republic of Korea

Telephone: +82-51-510-8475; Fax: +82-51-510-8437; E-mail: choibt@pusan.ac.kr

Figure S1. Effects of non-acupoint control stimulation in the Morris water maze test. Testing was conducted on day 10 post-BCAS induction. Mean time to locate the platform is indicated. EA, acupoints with electrical stimulation (electroacupuncture); NA, non-acupoints without electrical stimulation; NA-E, non-acupoints with electrical stimulation. Data expressed as the mean (±SEM). $^{###}P<0.001$ vs. sham control; $^{*}P<0.05$ vs. BCAS group.

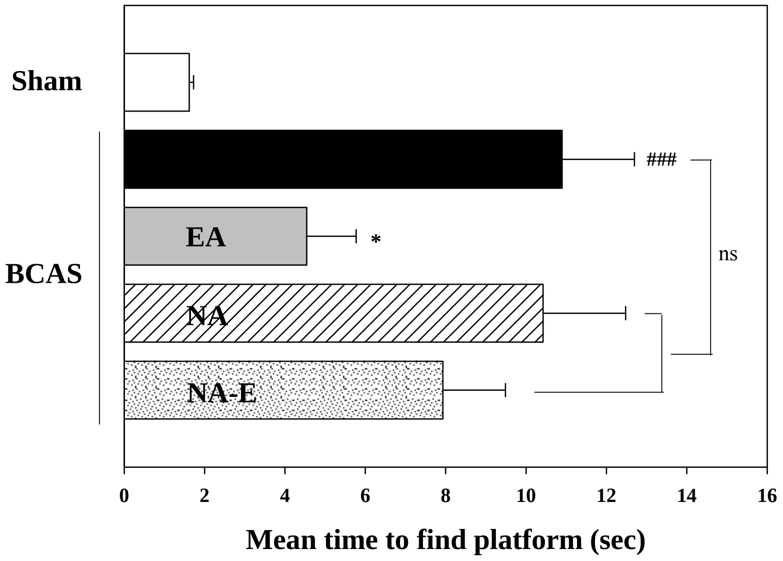

Figure S2. Morris water maze test. (A, C) Mean time to locate the platform. $^{###}P$<0.001 vs. sham control; $^{***}P$<0.001 vs. BCAS group. (B, D) Percent time spent in an annulus (20 cm diameter) around the platform location during probe trials. Data expressed as the mean (±SEM). $^{\$}P$<0.05 or $^{*}P$<0.05, and $^{**}P$<0.01 vs. sham control; $^{\#}P$<0.05, and $^{###}P$<0.001 vs. BCAS group.

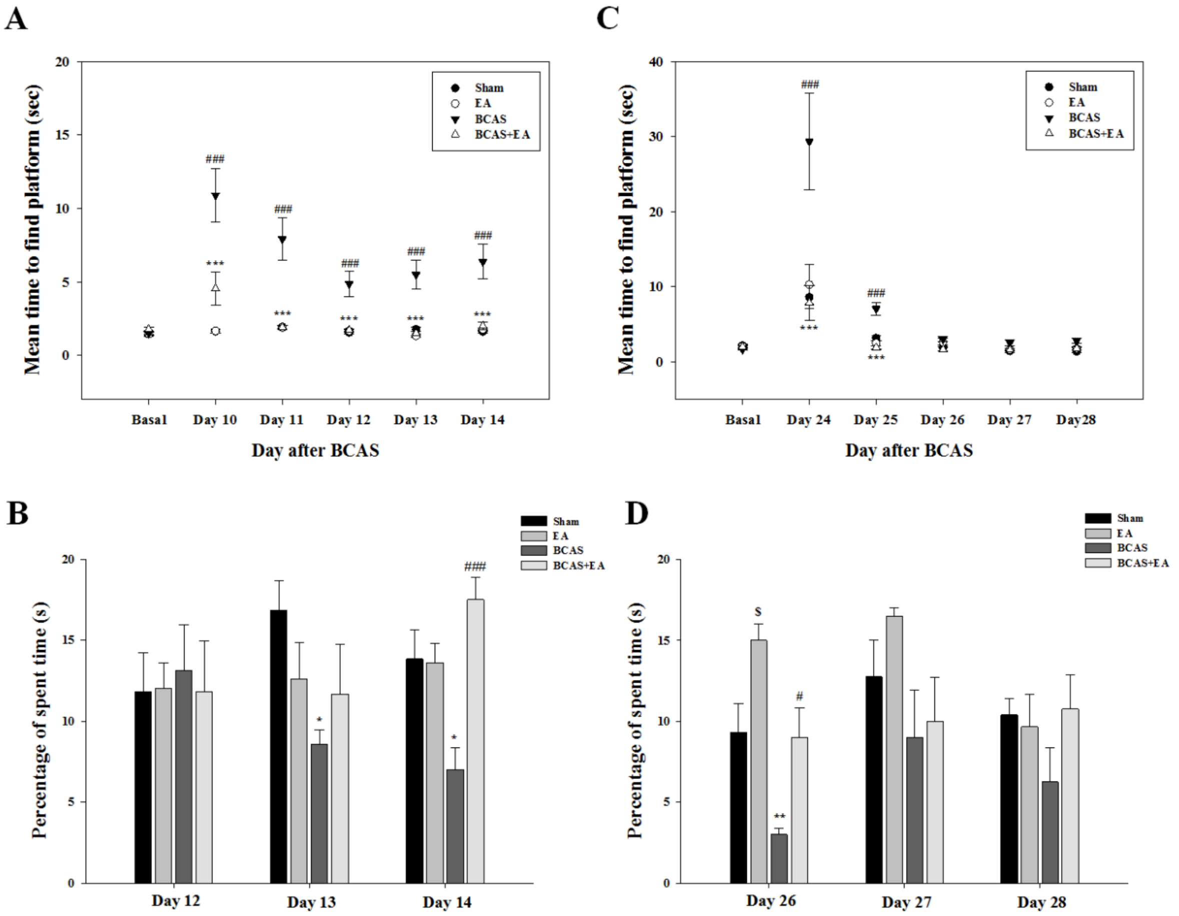

Figure S3. Immunofluorescent staining of Ki67 cells with PDGFRα in the subventricular zone. (A) Representative Ki67 staining images. Scale bar = 200 μm. (B) Representative Ki67 staining images with PDGFRα at days 14 and 18 post-BCAS induction. Scale bar = 50 μm.

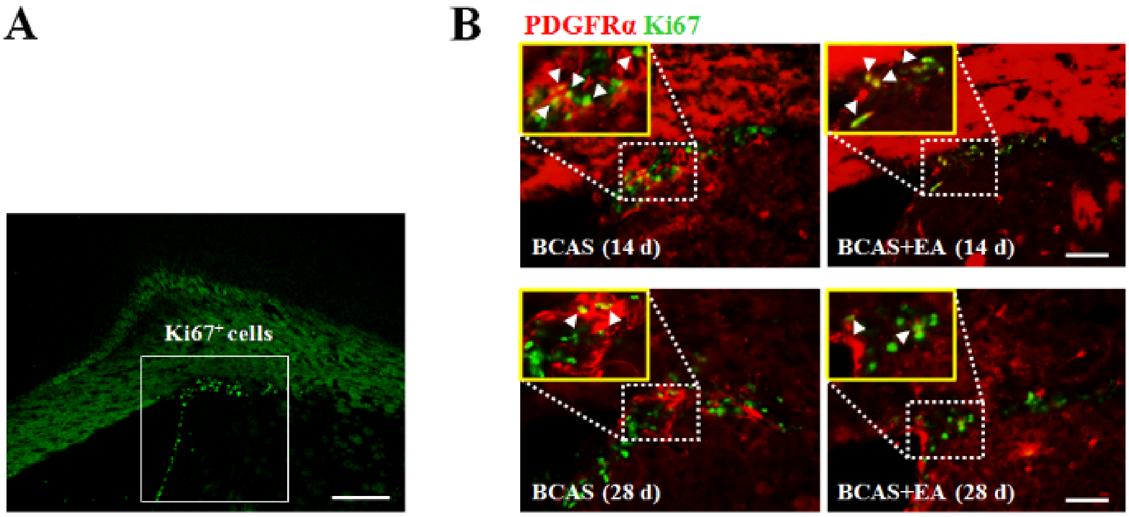

Figure S4. Double immunofluorescent staining for NT4/5 and NeuN, CNPase, or PDGFRα in the subventricular zone. (A, B) NT4/5-positive ($NT4/5^{+}$) cells and NT4/5 histogram analysis at days 14 and 28 post-BCAS induction. Data expressed as the mean (±SEM). $^{*}P<0.05$, $^{**}P<0.01$, and $^{***}P<0.001$ vs. sham control; $^{\#}P<0.05$ vs. EA group. Scale bar = 25 μm. (C) Representative NT4/5 staining images with NeuN, CNPase, and PDGFRα. Scale bar = 50 μm. (D, E) pTrkB-positive ($pTrkB^{+}$) cells and pTrkB histogram analysis at days 14 and 28 post-BCAS induction. Data expressed as the mean (±SEM). $^{***}P<0.001$ vs. sham control. (F) Representative pTrkB staining images with PDGFRα or CNPase (arrowheads). Scale bar = 25 μm.

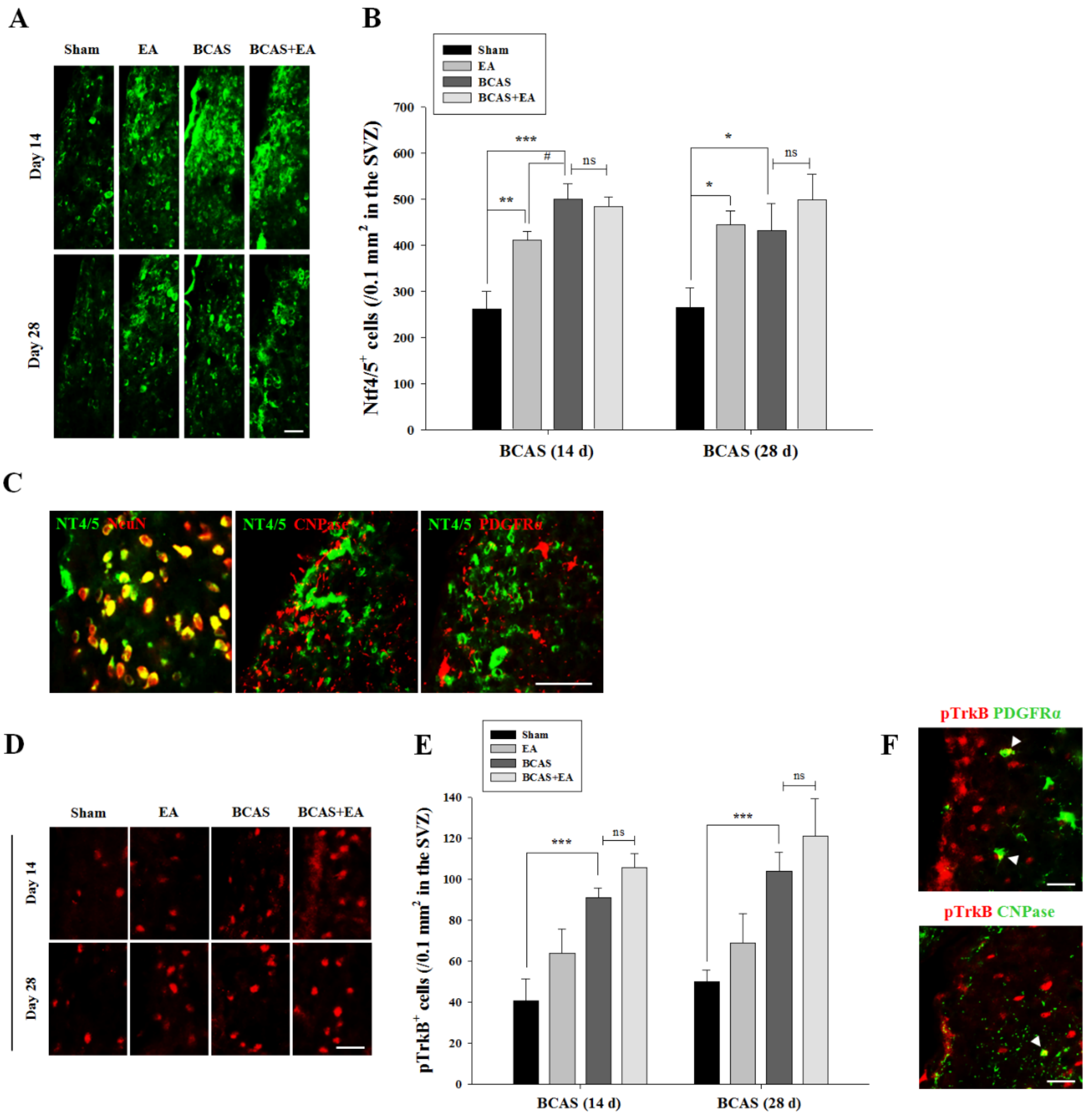

Supplement: Supplementary Information [file srep28646-s1.pdf]
